# Epidemiological, clinical, and public health response characteristics of a large outbreak of diphtheria among the Rohingya population in Cox’s Bazar, Bangladesh, 2017 to 2019: A retrospective study

**DOI:** 10.1371/journal.pmed.1003587

**Published:** 2021-04-01

**Authors:** Jonathan A. Polonsky, Melissa Ivey, Md. Khadimul Anam Mazhar, Ziaur Rahman, Olivier le Polain de Waroux, Basel Karo, Katri Jalava, Sirenda Vong, Amrish Baidjoe, Janet Diaz, Flavio Finger, Zakir H. Habib, Charls Erik Halder, Christopher Haskew, Laurent Kaiser, Ali S. Khan, Lucky Sangal, Tahmina Shirin, Quazi Ahmed Zaki, Md. Abdus Salam, Kate White

**Affiliations:** 1 World Health Organization, Geneva, Switzerland; 2 Institute of Global Health, Faculty of Medicine, University of Geneva, Geneva, Switzerland; 3 Médecins Sans Frontières, Amsterdam, the Netherlands; 4 World Health Organization Country Office for Bangladesh, Dhaka, Bangladesh; 5 Ministry of Health and Family Welfare, Dhaka, Bangladesh; 6 Global Outbreak Alert and Response Network (GOARN), Geneva, Switzerland; 7 Public Health England, London, United Kingdom; 8 London School of Hygiene and Tropical Medicine, London, United Kingdom; 9 UK-Public Health Rapid Support Team, London, United Kingdom; 10 Information Centre for International Health Protection (ZIG 1), Robert Koch Institute (RKI), Berlin, Germany; 11 World Health Organization South-East Asia Regional Office, New Delhi, India; 12 Epicentre, Paris, France; 13 Institute of Epidemiology Disease Control and Research (IEDCR), Dhaka, Bangladesh; 14 International Organization for Migration, Cox’s Bazar, Bangladesh; 15 College of Public Health, University of Nebraska Medical Center, Nebraska, United States of America; 16 World Health Organization Country Office for India, New Delhi, India; Johns Hopkins University Bloomberg School of Public Health, UNITED STATES

## Abstract

**Background:**

Unrest in Myanmar in August 2017 resulted in the movement of over 700,000 Rohingya refugees to overcrowded camps in Cox’s Bazar, Bangladesh. A large outbreak of diphtheria subsequently began in this population.

**Methods and findings:**

Data were collected during mass vaccination campaigns (MVCs), contact tracing activities, and from 9 Diphtheria Treatment Centers (DTCs) operated by national and international organizations. These data were used to describe the epidemiological and clinical features and the control measures to prevent transmission, during the first 2 years of the outbreak. Between November 10, 2017 and November 9, 2019, 7,064 cases were reported: 285 (4.0%) laboratory-confirmed, 3,610 (51.1%) probable, and 3,169 (44.9%) suspected cases. The crude attack rate was 51.5 cases per 10,000 person-years, and epidemic doubling time was 4.4 days (95% confidence interval [CI] 4.2–4.7) during the exponential growth phase. The median age was 10 years (range 0–85), and 3,126 (44.3%) were male. The typical symptoms were sore throat (93.5%), fever (86.0%), pseudomembrane (34.7%), and gross cervical lymphadenopathy (GCL; 30.6%). Diphtheria antitoxin (DAT) was administered to 1,062 (89.0%) out of 1,193 eligible patients, with adverse reactions following among 229 (21.6%). There were 45 deaths (case fatality ratio [CFR] 0.6%). Household contacts for 5,702 (80.7%) of 7,064 cases were successfully traced. A total of 41,452 contacts were identified, of whom 40,364 (97.4%) consented to begin chemoprophylaxis; adherence was 55.0% (*N* = 22,218) at 3-day follow-up. Unvaccinated household contacts were vaccinated with 3 doses (with 4-week interval), while a booster dose was administered if the primary vaccination schedule had been completed. The proportion of contacts vaccinated was 64.7% overall. Three MVC rounds were conducted, with administrative coverage varying between 88.5% and 110.4%. Pentavalent vaccine was administered to those aged 6 weeks to 6 years, while tetanus and diphtheria (Td) vaccine was administered to those aged 7 years and older. Lack of adequate diagnostic capacity to confirm cases was the main limitation, with a majority of cases unconfirmed and the proportion of true diphtheria cases unknown.

**Conclusions:**

To our knowledge, this is the largest reported diphtheria outbreak in refugee settings. We observed that high population density, poor living conditions, and fast growth rate were associated with explosive expansion of the outbreak during the initial exponential growth phase. Three rounds of mass vaccinations targeting those aged 6 weeks to 14 years were associated with only modestly reduced transmission, and additional public health measures were necessary to end the outbreak. This outbreak has a long-lasting tail, with Rt oscillating at around 1 for an extended period. An adequate global DAT stockpile needs to be maintained. All populations must have access to health services and routine vaccination, and this access must be maintained during humanitarian crises.

## Abstract 2

Jonathan Polonsky and colleagues report on a diphtheria outbreak among Rohingya people in Cox’s Bazar, Bangladesh during 2017-19.

## Author summary

**Why was this study done?:**

- Following the mass displacement of a highly vulnerable Rohingya population from Myanmar to Bangladesh in 2017, a large outbreak of diphtheria, a vaccine-preventable disease, occurred and spread rapidly, eventually lasting over 2 years. A large-scale international response effort was mounted to respond to the crisis, involving case isolation and treatment; tracing their close contacts and administering preventive antibiotic treatment; and mass vaccination campaigns (MVCs).
- Few, if any, previous studies have documented the epidemiological, clinical, and public health response characteristics of large outbreaks of diphtheria among vulnerable populations. We aim to provide such information to inform future public health response efforts.

**What did the researchers do and find?:**

- Between November 2017 and November 2019, 7,064 cases of diphtheria were identified. Most of the cases were among children, although a larger than expected proportion occurred among adults. Case fatality was low, with just 45 deaths.
- Symptoms were typical of diphtheria: sore throat (93.5%), fever (86.0%), pseudomembrane (34.7%), and gross cervical lymphadenopathy (GCL; 30.6%).
- Diphtheria antitoxin (DAT) was administered to 1,062 (89.0%) out of 1,193 eligible patients, with adverse reactions following among 229 (21.6%). A total of 41,452 household contacts were identified, of whom 40,364 (97.4%) consented to chemoprophylactic antibiotic therapy. An MVC achieved high coverage among the target population.

**What do these findings mean?:**

- High population density, poor living conditions, and fast growth rate caused explosive expansion of the outbreak during the initial exponential growth phase.
- Three rounds of mass vaccinations targeting children were associated with only modest reductions in transmission, and additional public health measures were necessary to end the outbreak.
- Diphtheria outbreaks in refugee settings may have a long-lasting tail and may require additional public health measures, such as expanded target age groups for vaccination, to bring to an end. Ensuring laboratory capacity for differential diagnosis in remote field sites is an important challenge.

## Introduction

On August 25, 2017, violence erupted in Rakhine state, Myanmar and resulted in the displacement of approximately 720,000 refugees—mostly stateless Rohingya—from Rakhine state into the neighboring district of Cox’s Bazar, Bangladesh. Together with previously displaced refugees, the total number of Rohingya in Bangladesh exceeds 930,000 [1].

Most of the recent Rohingya settled in preexisting and expanded camps in Ukhia and Teknaf areas or among the nearby host population. The speed and scale of displacement created a humanitarian emergency. Basic humanitarian services still remain under considerable strain despite intense efforts by the Bangladesh Government and medical and humanitarian partners working in the region [2]. The population is vulnerable to many diseases [1–3], and years of isolation and poor access to health services have resulted in low acquired or vaccine-derived immunity against a variety of diseases [4], leading to outbreaks of diphtheria, measles, and hepatitis A in 2017 and mumps and varicella in 2018, in Cox’s Bazar refugee camps [5–8]. Cases of Coronavirus Disease 2019 (COVID-19) have recently been detected in this population [9].

Diphtheria is a bacterial infection caused by toxigenic strains of *Corynebacterium diphtheriae*, primarily causing infection of the mucous membrane of the upper respiratory tract [10]. Local bacterial growth and subsequent tissue death may create a pseudomembrane potentially leading to airway obstruction; in severe cases, disseminated toxin may cause myocarditis or peripheral neuropathy [11,12]. The disease is typically transmitted through respiratory droplets or by direct contact with cutaneous lesions or fomites [11,12]. The incubation period is typically 2 to 5 days, and the infectious period for respiratory diphtheria is typically 2 to 4 weeks [12]. Diphtheria antitoxin (DAT) treatment is used to neutralize the circulating toxin in respiratory diphtheria, administered ideally 48 hours post-onset of symptoms [12–14], but due to the small risk for anaphylaxis [15], a sensitization test is frequently performed prior to administration. Antibiotic treatment is used to eliminate the bacteria, halt toxin production, and reduce transmissibility [11,12]. Prophylactic antibiotic treatment coupled with diphtheria toxoid vaccination should be considered for close contacts [11,12]. The inclusion of a diphtheria vaccine in childhood immunization protocols after World War II has reduced the incidence of diphtheria worldwide [16,17].

In settings where vaccination coverage is low, children under 15 years account for the highest proportion of diphtheria cases, but once high vaccination coverage has been achieved for some time, adolescents and adults account for the majority of cases, due to an absence of, or waning, immunity [18–21]. Case fatality ratios (CFRs) are 5% to 10%, with a higher CFR among young children [11,12,22]. Vaccination with at least 3 primary doses of diphtheria–tetanus–containing vaccine is recommended for persons aged 0 to 18 years, with at least a 6-month interval between second and third doses [15]. Three diphtheria toxoid–containing booster doses given after completion of the primary series, at 12 to 23 months, 4 to 7 years, and 9 to 15 years, is further recommended [15].

Despite a decrease in worldwide incidence, diphtheria outbreaks still occur, particularly among populations with poor vaccination coverage. Outbreaks in Central and South America, India and South Asia, Thailand, Laos, and Nigeria have been reported in recent decades [20,22–28]. In 2017, outbreaks occurred in Yemen, Venezuela, and Indonesia, with an ongoing outbreak in Haiti [29–32]. However, the Cox’s Bazar outbreak represents the largest outbreak since the 1990s, when over 140,000 cases were recorded in the Newly Independent States of the former Soviet Union [33].

On November 10, 2017, Médecins Sans Frontières (MSF) reported a case of suspected diphtheria in a 30-year-old Rohingya woman from Ukhiya. Additional suspected cases were reported, and after laboratory confirmation on December 4, 2017, the Bangladesh Ministry of Health and Family Welfare (MOH&FW) officially declared an outbreak. We describe the epidemiological and clinical features of the diphtheria outbreak among Rohingya and the local host population in Cox’s Bazar district, Bangladesh during the first 2 years of the ongoing outbreak, from November 10, 2017 to November 9, 2019, and the characteristics of the public health response to the outbreak.

## Methods

### Population, data source, and case definitions

All suspected cases were referred to 9 Diphtheria Treatment Centers (DTCs), operated by the Bangladesh Red Crescent, International Organization for Migration, MSF, or Samaritan’s Purse. DTCs collected daily data for demographic, epidemiological, and clinical characteristics of cases. Data were collected by field investigators using a standardized case investigation form which captured detailed sociodemographic characteristics, history of contact with known cases, and vaccination history. Additionally, close contacts, defined as household members (all persons sleeping in the same house/tent during the 5 nights prior to disease onset) and any persons with close contact (less than 1 meter) for a prolonged time (over 1 hour) during the 5 days prior to disease onset, and medical staff exposed to the oral or respiratory secretions of a case, were listed on a separate form [34]. Clinical and laboratory results were also recorded. All data were reported electronically to the World Health Organization’s (WHO) Early Warning, Alert and Response System (EWARS) for consolidation and cleaning [35]. We extracted case and contact tracing data for cases with symptom onset from November 10, 2017 to November 9, 2019. Reported cases were classified as suspected, probable, and confirmed cases on clinical and laboratory information, as per standard WHO and MOH&FW diphtheria surveillance case definitions:

- Confirmed: case patients reported as positive for toxigenic *C*. *diphtheriae* by PCR;
- Probable: case patients with an upper respiratory tract illness with laryngitis or nasopharyngitis or tonsillitis and at least one of the following signs: adherent membrane/pseudomembrane or gross cervical lymphadenopathy (GCL); and
- Suspected: any case with a clinical suspicion of diphtheria.

Due to the lack of specific and objectively observable signs and symptoms in the case definition for suspect cases, the admission, treatment, and reporting of suspect cases was therefore based on the subjective judgment of the clinician who examined the case.

### Clinical management

Patients were triaged upon arrival to the DTC based on disease severity in order to provide appropriate clinical management. According to the treatment protocol, DAT was to be administered to eligible patients with probable, clinical diagnosis of respiratory diphtheria, i.e., anyone presenting with either pseudomembrane or GCL. Further details on the protocol are reported elsewhere [34]. Owing to its limited global supply [15] and the need for close monitoring of patients during its administration, DAT was administered only to patients with severe disease requiring inpatient admission. DAT-treated patients were monitored closely for signs of respiratory distress from the development of airway obstruction or aspiration. Cardiac function was also monitored with ECG for conduction abnormalities and arrhythmias. In cases of upper airway obstruction, 2 ml of 1:1,000 solution nebulized adrenaline was administered hourly as a temporizing measure. Where shock due to heart failure was suspected, inotropes (such as dopamine or adrenaline) were administered.

Complications of diphtheria infection were assessed prior to case resolution (discharge or death), with long-term follow-up of patients performed up to 30 days post-discharge.

### Diagnostics

Material for laboratory diagnostics was obtained either by nasopharyngeal swab or by throat swab of the edges of the mucosal lesions, with extraction of pieces of membrane, and placed in Amies transport media. Further details on the protocol are reported elsewhere [34].

Swabs were cultured on Trypticase Soy Agar with 5% sheep blood (TSA+SB) (Thermo Fisher Scientific, Waltham, Massachusetts, United States of America) and/or Tinsdale agar (Remel, Lenexa, Kansas, USA) or BD (Franklin Lakes, New Jersey, USA). Isolates were identified with the API Coryne kit (bioMérieux, Durham, North Carolina, USA). Toxigenicity was determined by the modified Elek test [36]. Swabs underwent confirmatory laboratory testing for *tox*-bearing *C*. *diphtheriae* by real-time PCR with a multiplex PCR assay for the diphtheria toxin gene, *C*. *diphtheriae rpo*B and *Corynebacterium ulcerans/Corynebacterium pseudotuberculosis* (CUP) *rpo*B [37].

Initially, these tests were performed at the Institute of Epidemiology Disease Control and Research (IEDCR) in Dhaka; from March 2019 onwards, PCR analyses were performed at the IEDCR Field Laboratory in Cox’s Bazar Medical College Hospital. Between December 19 and December 25, 2017, during the peak of the outbreak, a representative subset of cases was tested to establish the sensitivity of the case definitions, and since June 2018, all suspected and probable cases were tested.

### Contact tracing, prophylaxis, and mass vaccination

Contact tracing was established in mid-December 2017. Close contacts of all probable and confirmed cases were identified during the case investigation process and targeted for vaccination, treatment, and follow-up over the course of prophylactic antibiotic treatment. Exposed contacts were vaccinated according to WHO strategy; pentavalent vaccine was administered for those aged 6 weeks to 6 years, while tetanus and diphtheria (Td) vaccine was administered to those aged 7 years and older. One booster dose was administered if documentary evidence of having completed the primary vaccination schedule was available, otherwise 3 doses were administered, with at least 4 weeks interval between doses. Further details on the protocol are reported elsewhere [34].

Different contact tracing and treatment regimen were implemented during 3 periods:

- Between establishment and February 20, 2018, a 7-day regimen of chemoprophylaxis was provided to contacts to self-administer, during which time contact tracers visited all contacts 3 times, on days 0, 3, and 7 after their last exposure with the case.
- Following a technical consultation among experts in the clinical management of diphtheria, from February 21 to May 5, 2018, the chemoprophylaxis course was reduced to a 3-day regimen, given to contacts to self-administer daily on days 0, 1, and 2, while contact tracers followed-up on days 0 and 3.
- Due to concerns about compliance and adherence to treatment, from May 6 onwards, chemoprophylaxis was provided to contacts by the contact tracers at visits on days 0, 1, and 2, who directly observed them taking the medication at this time. Contact tracers continued to make 1 final follow-up visit on day 7.

Pregnant women and children aged 6 weeks to 14 years among the Rohingya and host community residing in Ukhia and Teknaf Upazilas were targeted for 3 doses of vaccination over 3 rounds of a reactive diphtheria mass vaccination campaign (MVC) during December 12 to 31, 2017, January 27 to February 10, 2018, and March 10 to 25, 2018 [4]. Pentavalent vaccine against diphtheria, pertussis, tetanus, hepatitis B, and *Haemophilus influenzae* type b (Hib) was offered to children aged 6 weeks to 6 years and Td to children aged 7 to 14 years [2]. Administrative vaccination coverage for all rounds was estimated using vaccine consumption monitoring data collected during the campaigns with population estimates provided by MOH&FW and the United Nations High Commissioner for Refugees (UNHCR). Individual-level vaccination coverage was estimated by household survey and reported elsewhere [4].

### Statistical analysis

Continuous variables were described as median values with interquartile ranges (IQRs) and compared by *t* test or Mann–Whitney U-test. Frequencies for categorical variables were tabulated. The crude overall attack rate was calculated by dividing the total number of diphtheria-infected Rohingya by the settlement population. An adjusted estimate was made by restricting the analysis to confirmed and probable cases. We explored missing data for patterns of missingness and associations between missing and observed data; cases with missing data for variables of interest were excluded from analyses involving those variables.

We constructed unadjusted and adjusted multivariable Poisson regression models to identify risk factors for fatal outcome among confirmed and probable cases, expressed as risk ratios (RRs). To describe the role of diagnostics in this outbreak, we used Poisson regression to identify risk factors for testing PCR positive among all individuals tested (i.e., including those non-cases who tested negative and were excluded from other analyses).

To assess the validity of the probable case definition, we began by evaluating the sensitivity, specificity, and positive and negative predictive values (PPV and NPV, respectively) of presenting with either pseudomembrane or GCL against confirmatory laboratory testing as the reference. In order to identify practical enhancements for the clinical screening of patients in the absence of widespread testing, we assessed changes in the performance of the probable case definition through inclusion of additional signs and symptoms. Finally, we explored risk factors for presenting with either pseudomembrane or GCL using multivariable Poisson regression.

To assess the effectiveness of the active case finding and early case isolation response strategy, we calculated time from symptom onset to hospitalization, to which we fitted a multivariable linear regression model to identify risk factors for longer delays to presentation [38]. For response activity evaluations, we bisected the outbreak into 2 phases, corresponding to the periods before (period 1) and after (period 2) the end of the first round of the MVC (December 31, 2017). For age analyses, we divided cases into 3 age categories at the time of disease onset, based on vaccination schedule: under 7 years, 7 to 14 years, and 15 years and older. We calculated confidence intervals (CIs)of proportions assuming binomially distributed symptom occurrence. CFRs were calculated using observed deaths.

Daily growth rate (r) and epidemic doubling and halving times were estimated by Poisson regression of the incidence data during the growth and decay phases of the outbreak (bisected by the date of peak incidence by symptom onset), as implemented in the R package *R0* [39,40]. We parameterized the model with a serial interval of mean 4.5 days and standard deviation 6.0 days, derived from published literature [7,41–43]. The initial daily growth rate was used to estimate the initial reproduction ratio (R) [40]. As a sensitivity analysis, we also calculated r and R during the period that best fit an exponential growth curve, using the deviance R-squared statistic to automatically select the period [40].

We estimated the time-varying reproduction number (Rt) during the epidemic using dates of symptom onset, in a Bayesian framework using a Markov chain Monte Carlo approach calculated using a 7-day sliding window, as implemented in the R package *EpiEstim* [44–46].

Initial analyses were performed in real time to inform the outbreak response activities. An analysis plan was developed prior to analyzing the final dataset in November 2019 (S1 Analysis Plan), and no data-driven changes to this analysis took place, except where these informed the construction of stepwise regression models. All analyses were performed using R (version 4.0.2) [47].

### Ethics statement

The formulation of this work was discussed with national health institutes and the civil surgeon responsible for the oversight in the activities in the scope of the refugee crisis in Cox’s Bazar, Bangladesh. The data reported were collected in the course of the public health outbreak response activity; therefore no additional ethical approval was sought at the time of data collection. All individual case data were anonymized. This study is reported as per the Strengthening the Reporting of Observational Studies in Epidemiology (STROBE) guidelines (S1 Checklist).

## Results

### Descriptive epidemiology

**Fig 1:**
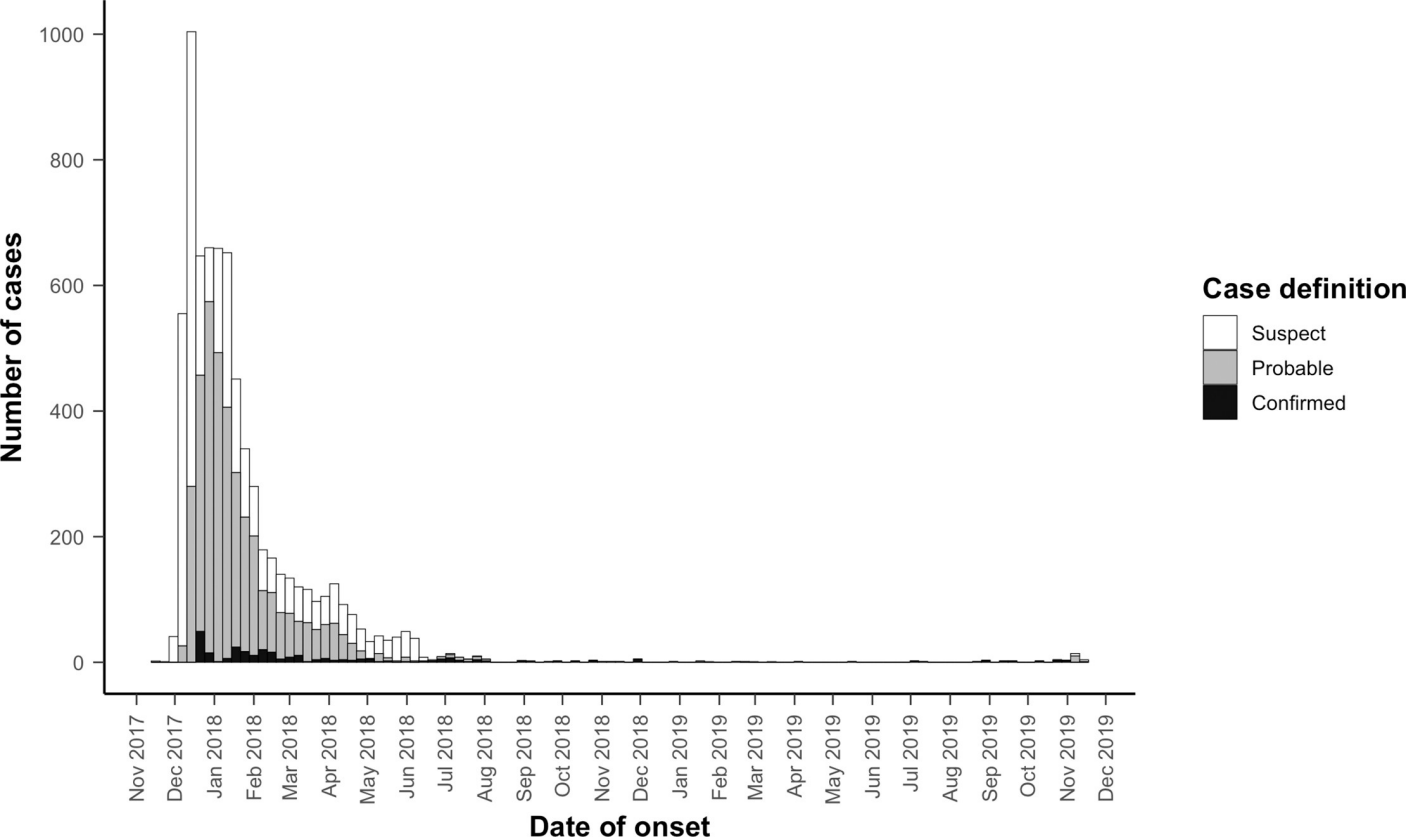
Weekly epidemic curve of diphtheria cases (confirmed, probable, and suspected) among Rohingya refugees, Cox’s Bazar, Bangladesh, November 10, 2017 to November 10, 2019.

A total of 7,064 cases with diphtheria were reported among Rohingya (and additional 227 cases were reported among the local Bangladesh population, but these will not be considered further in this analysis). There were 285 (4.0%) laboratory-confirmed, 3,610 (51.1%) probable, and 3,169 (44.9%) clinically suspected cases (Table 1, Fig 1), with an additional 1,624 suspected cases testing negative for diphtheria. The crude diphtheria attack rate over the first 2 years of the outbreak was 51.5 cases per 10,000 person-years; including only confirmed and probable cases, the attack rate was 28.4 cases per 10,000 person-years. The median age of cases was 10 years (IQR 7 to 15; range 0 to 85), with those aged under 7 years, 7 to 14 years, and 15 years and older representing 1,729 (24.5%), 3,309 (46.8%), and 2,026 (28.7%) of cases, respectively (Table 1). Overall, 3,126 (44.3%) were male. There were marked differences in the sex distribution between age groups; although the ratio of females to males was approximately equal to 1 (0.98) among children aged under 15 years (2,488 females:2,550 males), that among adult cases was 2.5 (1,450 females:570 males). Most patients (5,850, 82.8%) recovered, while 45 were recorded as having died from diphtheria: 2 among confirmed, 19 among probable, and 24 among suspected cases (overall CFR 0.6% with no significant difference by case definition) (Table 2). A total of 1,075 cases (15.2%) were lost to follow-up (no final outcome recorded) following an initial evaluation. We found no evidence of patterns of missingness or associations between missing and observed data.

**Table 1:** Characteristics of diphtheria cases by case definition among Rohingya refugees, Cox’s Bazar, Bangladesh, November 10, 2017 to November 10, 2019.

		Case definition			
Characteristic		Confirmed	Probable	Suspect	Total
**Age**	**<7**	59	873	797	1,729
	**7–14**	136	1,743	1,430	3,309
	**15–29**	83	784	693	1,560
	**30–44**	4	158	170	332
	**45+**	3	48	77	128
	**Missing**	0	4	2	6
**Sex**	**Male**	118	1,632	1,376	3,126
	**Female**	167	1,978	1,793	3,938
**Total**	**-**	285	3,610	3,169	7,064

**Table 2:** Number (%) of signs and symptoms, complications, and treatment outcomes among diphtheria cases, by case definition, among Rohingya refugees, Cox’s Bazar, Bangladesh, November 10, 2017 to November 10, 2019.

Characteristic		Confirmed (*N* = 285)	Probable (*N* = 3,610)	Suspected (*N* = 3,169)	All cases (*N* = 7,064)
**Sign/symptom**	**Sore throat**	282 (98.9)	3,551 (98.4)	2,771 (87.4)	6,604 (93.5)
	**Fever**	259 (90.9)	3,246 (89.9)	2,573 (81.2)	6,078 (86.0)
	**Pseudomembranes**	195 (68.4)	2,258 (62.5)	0	2,453 (34.7)
	**Difficulty in swallowing**	183 (64.2)	1,269 (35.2)	1,026 (32.4)	2,478 (35.1)
	**Lymphadenopathy**	125 (43.9)	2,040 (56.5)	0	2,165 (30.6)
	**Tonsillitis**	45 (15.8)	284 (7.9)	154 (4.9)	483 (6.8)
	**Nasal regurgitation**	15 (5.3)	159 (4.4)	208 (6.6)	382 (5.4)
	**Nasal blood**	13 (4.6)	34 (0.9)	41 (1.3)	88 (1.2)
	**Lethargy**	2 (0.7)	39 (1.1)	45 (1.4)	86 (1.2)
**Complications**	**Neuropathy**	4 (1.4)	21 (0.6)	3 (0.1)	28 (0.4)
	**Respiratory distress**	3 (1.1)	19 (0.5)	4 (0.1)	26 (0.4)
	**Cutaneous necrosis**	1 (0.4)	2 (0.1)	1 (<0.1)	4 (0.1)
	**Irregular heart rhythm**	0 (0.0)	3 (0.1)	2 (0.1)	5 (0.1)
	**Shock**	0 (0.0)	5 (0.1)	1 (0.1)	6 (0.1)
	**Kidney damage**	0 (0.0)	2 (0.1)	0 (0.0)	2 (<0.1)
**Treatment outcomes**	**Recovered**	241 (84.6)	3,143 (87.1)	2,466 (77.8)	5,850 (82.8)
	**Death**	2 (0.7)	19 (0.5)	24 (0.8)	45 (0.6)
	**Transferred**	2 (0.7)	53 (1.5)	29 (0.9)	84 (1.2)
	**Left against medical advice**	2 (0.7)	72 (2.0)	106 (3.3)	180 (2.5)
	**Lost to follow-up**	38 (14.0)	317 (8.8)	540 (17.0)	895 (12.7)
	**Other**	0 (0.0)	6 (0.2)	4 (0.1)	10 (0.1)

**Fig 2:**
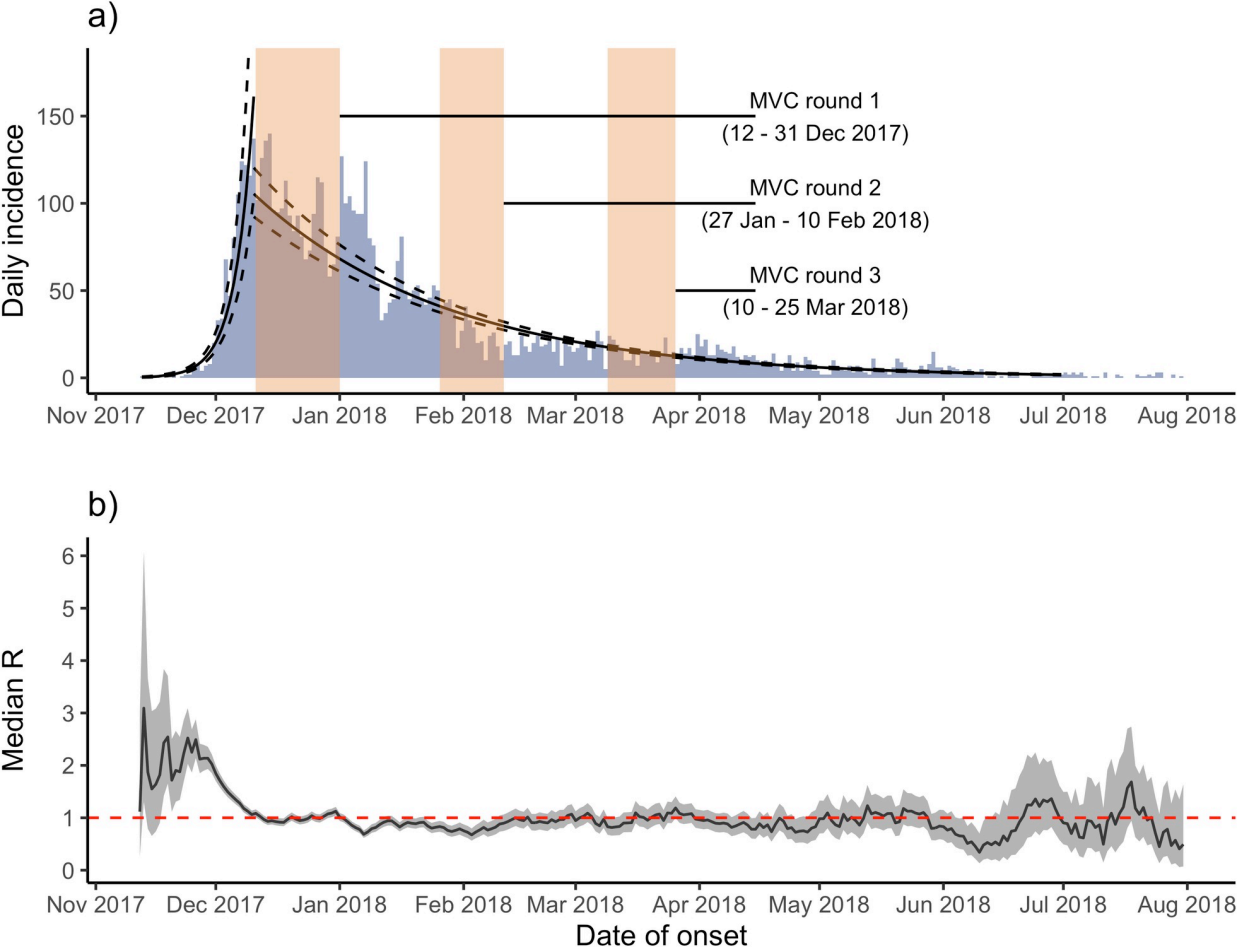
a) Poisson models fitted to daily incidence over time, bisected by date of peak incidence (December 14, 2017), Cox’s Bazar, Bangladesh, November 10, 2017 to July 31, 2018. The timing of 3 rounds of MVC are shown as transparent beige bars. b) Estimates of time-varying reproduction number (Rt) for diphtheria cases, Cox’s Bazar, Bangladesh, November 10, 2017 to July 31, 2018. The time period was limited to July 31, 2018 owing to highly unstable estimates of Rt produced thereafter due to the effect of sporadic cases. MVC, mass vaccination campaign.

We estimated *r* as 0.16 per day (95% CI: 0.15 to 0.17) and R as 2.1 (95% CI: 2.0 to 2.1) during the period before peak incidence (December 14, 2017). Epidemic doubling and halving times were calculated as 4.4 days (95% CI 4.2 to 4.7) and 32.9 days (95% CI: 31.9 to 33.9), respectively (Fig 2A). The sensitivity analysis identified the period best describing exponential growth as November 13 and December 7, 2017, during which time R was estimated as 3.0 (95% CI 2.8 to 3.3). An additional sensitivity analysis restricted to confirmed and probable cases only estimated *r* of 0.11 per day (95% CI: 0.10 to 0.12), R of 1.7 (95% CI: 1.7 to 1.8), and doubling and halving times of 6.3 days (95% CI 5.9 to 6.8) and 25.6 days (95% CI: 24.6 to 26.7), respectively.

The MVC launch coincided with a decrease in incidence and transmissibility, as measured by Rt (Fig 2). As is frequently observed, Rt was high and unstable in the early stage of the epidemic when the epidemic was growing exponentially but the overall epidemic size was low, subsequently decreasing rapidly, crossing the threshold of Rt = 1.0 around the date of peak incidence (Fig 2B). During this initial phase, median Rt was 2.62 (95% CI: 1.95 to 3.57), after which Rt oscillated around 1 (median Rt = 0.96, 95% CI: 0.71 to 1.30) for an extended period.

### Clinical management

The overall median delay from illness onset to examination was 2 days (IQR: 1 to 3, range: 0 to 33 days), decreasing significantly from 2.9 days in period 1 to 2.3 days in period 2 (t(5548) = 10.8, *p* < 0.001). Overall, one-quarter of cases reported receiving at least 1 dose of diphtheria toxoid vaccine (1640, 23.2%), increasing markedly from 1.0% in period 1 to 51.1% in period 2. Of those vaccinated, 524 (32.0%) were vaccinated prior to symptom onset, and 54 (3.3%) were vaccinated after symptom onset (data missing for 1,062 (64.8%) patients). Details of signs and symptoms at the time of admission to DTC were available for 6,730 (95.3%) of cases. These included sore throat (93.5%), fever (86.0%), pseudomembrane (34.7%), difficulty swallowing (35.1%), and GCL (30.6%) (Table 2). Altogether, 73 (1.0%) of the probable or confirmed cases reported complications prior to discharge or death; peripheral neuritis/neuropathy (*n* = 28, 38.4%) and respiratory distress (*n* = 26, 35.6%) were reported (Table 2). Out of 949 patients for whom follow-up was done at 30 days post-discharge, 1 patient was recorded as having died due to an unrelated cause, and one was recorded as having recovered with sequelae (no additional information recorded).

Antibiotic treatment was received by 4,379 (62.0%) patients: 4,230 (59.9%) received azithromycin, 67 (0.9%) received penicillin, and 3 (0.04%) received erythromycin. A total of 183 (2.6%) patients received an unspecified “other” antibiotic, while 220 (3.1%) received a steroid treatment.

DAT was administered to 1,062 (89.0%) out of 1,193 eligible patients, of whom 1,023 (96.3%) also received antibiotic treatment. No differences in age group or sex were observed between those DAT-eligible patients who received DAT and those who did not, but patients presenting during period 1 were less likely than those presenting during period 2 to have been administered DAT (78.5% versus 92.0%, *X*^*2*^ (1, *N* = 1044) = 19.0, *p* < 0.001). Despite protocols to perform DAT sensitivity tests on all eligible patients prior to administration, this was only done for 545 (52.3%), of whom 40 (7.3%) were hypersensitive. Adverse reactions following DAT administration were reported among 229 (21.6%) patients who received DAT, of whom 65 (28.1%) and 4 (1.7%) had febrile and anaphylactic reactions, respectively. As a preventive measure against side effects, supportive treatment of antihistamine and/or corticosteroids during DAT administration was given to 754 (77.5%) of DAT-treated patients.

The median delay from symptom onset to presentation at health facilities was significantly greater among fatal cases (median delay 4 [IQR 2 to 6] days) compared to survivors (median delay 2 [IQR 1 to 3] days), *p* < 0.001, and the risk of dying increased substantially for each additional day of delay (Table 3). In univariable analyses, confirmed and probable cases aged 0 to 6 years had the greatest risk of death, but there was no association observed for sex. Cases presenting with respiratory distress (RR = 13.21 [95% CI 4.32 to 33.73]) or pseudomembrane (RR = 11.76 [95% CI 2.45 to 211.01]) were more likely to be fatal. Cases presenting with GCL were less likely to be fatal (RR = 0.32 [95% CI 0.11 to 0.79]). Patients who received antibiotic treatment were less likely to die (RR = 0.38 [95% CI 0.16 to 0.89]); receiving DAT was associated with a higher risk of death (RR = 3.35 [95% CI 1.02 to 14.93]). No fatal cases were vaccinated prior to disease onset. In a multivariable model containing all these explanatory variables, younger age, respiratory distress, presenting with pseudomembrane, and treatment with DAT remained strongly associated with higher risk of death, while antibiotic treatment remained a significant protective factor (Table 3). There was no evidence of an effect on risk of death of receiving the full “package” of interventions (vaccination and antibiotic treatment and DAT treatment) versus receiving none of the package (RR = 0.36 [95% CI 0.04 to 3.04]), but with just 2 deaths in each group, the study was likely underpowered to adequately explore this relationship.

**Table 3:** Multivariable Poisson regression for predictors of fatality among for confirmed and probable diphtheria patients, Cox’s Bazar, Bangladesh, November 10, 2017 to November 10, 2019.

			Unadjusted		Adjusted	
Independent variable	Level	*N*	RR (95% CI)	*p*-value	RR (95% CI)	*p*-value
**Age group**	**0–6**	932	Reference	-	Reference	-
	**7–14**	1,879	0.02 (0.001–0.003)	<0.001	0.11 (0.02–0.53)	0.006
	**15+**	1,080	0.00 (0.00–Inf)	0.993	0.00 (0.00–Inf)	0.992
**Sex**	**Female**	2,145	Reference	-	-	-
	**Male**	1,750	0.75 (0.30–1.79)	0.530	-	-
**Vaccination status**	**Unvaccinated**	1,214	Reference	-	-	-
	**Vaccinated**	845	1.08 (0.21–4.89)	0.922	-	-
**Delay**	1 day^*^	3,895	1.13 (0.99–1.22)	0.011	1.13 (0.99–1.29)	0.076
**Respiratory distress**	**False**	3,805	Reference	-	Reference	-
	**True**	90	13.21 (4.32–33.73)	<0.001	13.65 (4.59–40.61)	<0.001
**Pseudomembrane**	**False**	1,442	Reference	-	Reference	-
	**True**	2,453	11.76 (2.45–211.01)	0.016	10.85 (1.28–92.27)	0.029
**GCL**	**False**	1,730	Reference	-	Reference	-
	**True**	2,165	0.32 (0.11–0.79)	0.018	0.50 (0.17–1.47)	0.207
**Antibiotic treatment**	**False**	1,141	Reference	-	Reference	-
	**True**	2,754	0.38 (0.16–0.89)	0.025	0.22 (0.06–0.76)	0.017
**DAT**	**False**	980	Reference	-	Reference	-
	**True**	983	3.35 (1.02–14.93)	0.066	5.43 (1.39–21.23)	0.015

* RR for each additional day delay between disease onset and presentation at health facility.

Covariates with a *p*-value of < = 0.1 were included in the multivariate model.

CI, confidence interval; DAT, diphtheria antitoxin; GCL, gross cervical lymphadenopathy; RR, risk ratio.

### Diagnostics

Multiplex PCR assay was performed on 1,329 cases, and 271 (20.4%) tested positive for *tox*-bearing *C*. *diphtheriae*. Additionally, 659 swabs were cultured. Among 705 laboratory-tested pseudomembrane positive cases, only 215 (30.5%) were PCR positive. Cases aged 15 years and older were less likely to be laboratory confirmed than younger cases. Cases presenting with a pseudomembrane or with symptoms onset during the pre-vaccination campaign period were more likely to be confirmed (Table 4). Owing to very limited laboratory capacity to respond to the large demand at the time, cases that tested diphtheria negative were not investigated further.

**Table 4:** Multivariable Poisson regression for predictors for diphtheria patients testing positive (as opposed to negative) for toxigenic *C*. *diphtheriae* strain by PCR, Cox’s Bazar, Bangladesh, November 10, 2017 to November 10, 2019.

			Unadjusted		Adjusted	
Independent variable	Level	*N*	RR (95% CI)	*p*-value	RR (95% CI)	*p*-value
**Age group**	**0–6**	1,942	Reference	-	Reference	-
	**7–14**	3,968	0.88 (0.66–1.19)	0.393	0.95 (0.69–1.32)	0.751
	**15+**	2,971	0.44 (0.32–0.60)	<0.001	0.52 (0.37–0.74)	<0.001
**Sex**	**Female**	5,124	Reference	-	Reference	-
	**Male**	3,764	1.34 (1.07–1.67)	0.011	1.12 (0.88–1.43)	0.361
**Vaccination status**	**Unvaccinated**	2,097	Reference	-	-	-
	**Vaccinated**	2,211	0.82 (0.61–1.10)	0.177	-	-
**Period**	**Pre-campaign**	3,091	Reference	-	Reference	-
	Campaign era^*^	5,205	0.41 (0.32–0.55)	<0.001	0.63 (0.47–0.84)	0.001
**Pseudomembrane**	**False**	5,856	Reference	-	Reference	-
	**True**	2,981	3.93 (3.11–4.99)	<0.001	3.78 (2.95–4.88)	<0.001
**GCL**	**False**	5,820	Reference	-	-	-
	**True**	3,017	0.91 (0.73–1.14)	0.443	-	-

* Patients were divided into 2 periods, according to the date of symptom onset relative to the completion of the first round of MVC.

Patients not tested or those with indeterminate results were excluded.

Covariates with a *p*-value of < = 0.1 were included in the multivariate model.

CI, confidence interval; GCL, gross cervical lymphadenopathy; MVC, mass vaccination campaign; RR, risk ratio.

The probable case definition—presenting with pseudomembrane or GCL—was 83.9% sensitive and 34.4% specific, with 19.3% PPV and 91.9% NPV (Table 5). Sensitivity was enhanced by the addition of either sore throat (99.2%) or fever (98.4%) to this case definition, although specificity was greatly reduced as a result. Conversely, specificity was enhanced by the addition of nasal regurgitation (93.3%), lethargy (98.1%), or nasal blood (98.5%), the latter also providing the most balanced performance in terms of PPV (70.9%) and NPV (86.8%).

**Table 5:** Sensitivity, specificity, PPV, and NPV of an enhanced probable case definition (presence of either PM and/or GCL) with inclusion of additional signs and symptoms, Cox’s Bazar, Bangladesh, November 10, 2017 to November 10, 2019.

Additional sign/symptom	*n* (*N* = 2006)	Sensitivity (%)	Specificity (%)	PPV (%)	NPV (%)
**None (PM and/or GCL alone)**	1,373	83.9	34.4	19.3	91.9
**Sore throat**	1,367	99.2	0.5	15.7	79.3
**Fever**	1,304	98.4	1	15.7	76.9
**Difficulty in swallowing**	1,136	87.9	8.2	15.2	78.4
**Tonsillitis**	396	48.4	59.3	18.2	86
**Nasal regurgitation**	52	20.3	93.3	36.1	86.2
**Nasal blood**	21	20	98.5	70.9	86.8
**Lethargy**	13	3.8	98.1	27.7	84.5

GCL, gross cervical lymphadenopathy; NPV, negative predictive value; PM, pseudomembrane; PPV, positive predictive value.

The risk of presenting with pseudomembrane was greater among children aged 7 to 14 years (RR = 1.42 [95% CI 1.19 to 1.71]) and among adults aged 15 years or older (RR = 1.66 [95% CI 1.38 to 2.02]) (compared to children aged under 7 years) and lower among vaccinated cases (with a dose response such that risk was lowest among those having received all 3 doses, RR = 0.64 (95% CI 0.50 to 0.82]) and patients with symptom onset during period 2 (RR = 0.54 [95% CI 0.45 to 0.65]) (Table 6). There was no association observed between sex of the patient and presenting with pseudomembrane. By contrast, the risk of presenting with GCL was greater among children aged under 7 years, and among males (RR = 1.11 [95% CI 1.00 to 1.24]), but as with pseudomembrane, the risk was lowest among vaccinated cases having received all 3 doses (RR = 0.68 (95% CI 0.56 to 0.83]) and those presenting during period 2 (RR = 0.79 (95% CI 0.66 to 0.93]).

**Table 6:** Multivariable Poisson regression for risk factors for diphtheria patients presenting with pseudomembrane and lymphadenopathy, Cox’s Bazar, Bangladesh, November 10, 2017 to November 10, 2019.

			Presence of pseudomembrane				Presence of GCL			
			Unadjusted		Adjusted		Unadjusted		Adjusted	
Independent variable	Level	*N*	RR (95% CI)	*p*-value	RR (95% CI)	*p*-value	RR (95% CI)	*p*-value	RR (95% CI)	*p*-value
**Age group**	**0–6**	1,729	Reference	-	Reference	-	Reference	-	Reference	-
	**7–14**	3,309	1.28 (1.15–1.42)	<0.001	1.42 (1.19–1.71)	<0.001	0.93 (0.84–1.03)	0.165	0.96 (0.85–1.10)	0.569
	**15+**	2,020	1.42 (1.27–1.59)	<0.001	1.66 (1.38–2.02)	<0.001	0.71 (0.63–0.80)	<0.001	0.69 (0.59–0.80)	<0.001
**Sex**	**Female**	3,938	Reference	-	-	-	Reference	-	Reference	-
	**Male**	3,126	0.93 (0.86–1.01)	0.100	-	-	1.16 (1.07–1.27)	<0.001	1.11 (1.00–1.24)	0.052
**Vaccination doses**	**0 (ref.)**	1,876	Reference	-	Reference	-	Reference	-	Reference	-
	**1**	510	0.67 (0.55–0.81)	<0.001	0.77 (0.62–0.94)	0.013	1.00 (0.86–1.15)	1.000	0.99 (0.85–1.16)	0.947
	**2**	493	0.72 (0.59–0.87)	0.001	0.87 (0.71–1.06)	0.172	1.06 (0.91–1.22)	0.452	1.00 (0.85–1.16)	0.962
	**3**	382	0.54 (0.42–0.68)	<0.001	0.64 (0.50–0.82)	0.001	0.71 (0.58–0.85)	<0.001	0.68 (0.56–0.83)	<0.001
**Period**	**Pre-campaign**	2,927	Reference	-	Reference	-	Reference	-	Reference	-
	Campaign era^*^	3,617	0.77 (0.71–0.83)	<0.001	0.54 (0.45–0.65)	<0.001	2.27 (2.06–2.51)	<0.001	0.79 (0.66–0.93)	0.006

* Patients were divided into 2 periods, according to the date of symptom onset relative to the completion of the first round of MVC.

Covariates with a *p*-value of < = 0.1 were included in the multivariate model.

CI, confidence interval; GCL, gross cervical lymphadenopathy; MVC, mass vaccination campaign; RR, risk ratio.

### Contact tracing, prophylaxis, and reactive and mass vaccination

Household contacts for 5,702 (80.7%) of 7,064 cases were successfully traced. A total of 41,452 contacts were identified (median 5 contacts per case, range 0 to 49), of whom 40,364 (97.4%) consented to begin chemoprophylaxis; adherence was 55.0% (*N* = 22,218) at 3-day follow-up. The proportion of household contacts vaccinated was 64.7% overall. The administrative coverage of the MVC was 88.5%, 110.4%, and 104.0% for the first, second, and third rounds, respectively. Data on the proportion of contacts and vaccinated individuals who later developed disease were unavailable.

## Discussion

### Main findings

To our knowledge, this outbreak was the largest reported diphtheria outbreak occurring among refugees. It reflects a long history of under-provision of health services to the Rohingya in Rakhine state, including routine vaccinations. Very low vaccination coverage, high population density, and poor living conditions were associated with a doubling time of around 4 days was observed, with explosive expansion of the outbreak during the initial exponential growth phase. Our findings suggest that 3 rounds of mass vaccinations targeting all individuals aged 6 weeks to 14 years and pregnant women in the affected areas may have contributed to reducing transmission, but alone were not sufficient to end the outbreak. Indeed, this outbreak had a long-lasting tail, with Rt oscillating at around 1 for an extended period of time. Cases continue to appear with case clusters still occurring, likely reflecting the lack of universal vaccination coverage [4,48], which may enable transmission to continue [11,49]. Concurrent diphtheria outbreaks in Yemen, Indonesia, Venezuela, and Haiti led to shortages of DAT availability globally [7,32,50], potentially further hampering the outbreak response.

Approximately half of the cases were probable, with only a small fraction of cases test positive. Overall, two-thirds of the cases were children <15 years of age. The age distribution, with older cases than expected for a disease that typically affects young children [19], likely reflects low diphtheria vaccination coverage in this population, or waning immunity, especially among adults. Similar findings have been reported for diphtheria [51,52] and measles [53,54] among refugees and other populations lacking adequate routine vaccine coverage. The female predominance among adults might reflect the practice of nursing of small infants by female household members—a similar effect was observed in a large outbreak in the former Soviet Union [33]—but may also reflect health-seeking behavior or the demographic structure of the refugee population. The majority of Rohingya cases (90%) reported to be unvaccinated, consistent with other recent diphtheria outbreaks reported from South Africa [55], Lao People’s Democratic Republic [28], Nigeria [22], and Colombia [26]). The clinical presentation of the cases in the current outbreak was typical of diphtheria [56], yet the mortality was low. Immediate complications were rare.

While penicillin or erythromycin are recommended for treatment, oral azithromycin, usually reserved for penicillin-sensitive patients [57], was the most frequently used antibiotic; oral azithromycin is a once daily dose, which was judged more feasible in this limited resource setting than oral penicillin and erythromycin, which require administration up to 4 times daily. This did not appear to have a negative impact on clinical outcomes; indeed, we observed a low CFR, and low clinical severity in general, during this outbreak relative to those previously described [43]. One explanation could be a combination of the following:

1. the implementation of an active case finding strategy leading to shorter delays between disease onset and presentation and higher reporting rates among mild cases; and
2. effective treatment provided by the national and international healthcare professionals surged to respond to this outbreak [58,59].

The deaths occurred more commonly among children, and longer delays between onset and admission were associated with increased risk of death, highlighting the importance of adequate medical attention in the initial stages of diphtheria. The low observed CFR may also partially be due to low PPV of the case definitions, such that a substantial proportion of cases may in fact have been diseases with similar clinical presentation to diphtheria, e.g., bacterial tonsillitis. Our finding that presenting with GCL was associated with a lower risk of death suggests that this clinical sign, used in the probable case definition, may have been frequently misdiagnosed or associated with other less severe diseases. However, as low CFR was also observed among confirmed and probable cases, another possible explanation is co-circulation of less virulent isolates of diphtheria [57] and of diphtheria-like illness (which might have led to extensive false-positive probable case classification). Such information was unavailable here, and this lack of real-time information on the genotypes of the outbreak strains, the value of which has been demonstrated elsewhere [60], was identified to be limiting a comprehensive understanding of the disease in this particular setting [61].

The risk of presenting with pseudomembrane and GCL was greater among those detected during period 1, indicating either under-ascertainment of mild cases during the early phase of the epidemic, decreased severity post-vaccination, or that the case definition was implicitly expanded by clinicians to be more sensitive and less specific during the later periods of low incidence.

Respiratory distress and treatment with DAT were associated with greater risk of death, presumably reflecting more severe disease among these patients. Antimicrobial treatment was associated with a lower risk of death, suggesting it was an effective treatment.

We estimated the basic reproduction number R0 during the exponential growth phase at 3.0, which approximates to that reported in a pooled analysis of previous outbreaks of diphtheria of 2.7 [95% CI 1.7 to 4.3] [43]. The findings that both transmissibility and the delay from disease onset to presentation at health facilities decreased during the early phase of this outbreak suggest that implemented interventions, specifically contact tracing, active case finding, and early isolation of cases, were effective. The high vaccination campaign coverage rates reflected high overall vaccination campaign coverage estimates of 93% for 1 dose and 89% for 2 doses, as reported in a vaccination coverage and seroprevalence survey conducted approximately 1 month after the final round of the vaccination campaign [4]. However, this declined to just 77% for a third dose.

### Strengths and limitations

Lack of adequate diagnostic capacity was a major limitation of this study, with a majority of cases unconfirmed and no clear understanding of the range of co-circulating diphtheria-like illnesses. Therefore, the proportion of true diphtheria cases remained unknown, potentially biasing the results. Additionally, the tox+ isolates were not systematically tested using Elek test for the production of toxin, which would have informed the understanding of the circulating strains and their toxicity and the extent of misdiagnosis. Introduction of standardized case report forms, implementation of the EWARS-in-a-box system, and extensive retrospective chart review largely resolved data quality concerns. The limitations on beds, staff, DAT, vaccine, and antibiotic supply on-site potentially hampered control efforts during this outbreak. Despite these limitations, our analyses were informed by a large study sample size due to the unprecedented size of the outbreak. This article may provide a generalizable description of diphtheria outbreaks, especially under crowded and inadequate hygienic conditions.

## Implications for further research, clinical practice, and public policy

Only approximately 20% of those tested for diphtheria tested positive in this population. As a large majority of pseudomembrane-positive cases (considered diagnostically highly accurate of diphtheria infection) tested negative, the validity of the test and sampling procedure should be evaluated. The diphtheria toxin gene target in the PCR used is sensitive to approximately 10 genome copies per reaction, while the rpoB targets for *C*. *diphtheriae* and *C*. *ulcerans/C*. *pseudotuberculosis* are somewhat less sensitive, between 10 and 100 genome copies per reaction [37]. While clinical validation is unavailable to provide sensitivity data in detail, this PCR assay was reported to be more sensitive than a previous assay during an analysis of 105 clinical samples [37]. False-negative test results may have arisen if specimen quality was poor, if there were delays in testing, or if patients received antibiotics (as contacts or for treatment) prior to testing [62]. Ultimately, the low proportion of cases receiving a diagnostic test and co-circulation of illnesses that resemble diphtheria indicates the need for improvement in alternative diagnostics and categorization of suspect cases, including the use of mobile and local laboratory capacity and deployment of rapid diagnostics tests for diphtheria. Our evaluation of the validity of the probable case definition (presence of pseudomembrane or GCL) suggested that it was relatively high in sensitivity but quite unspecific, with high NPV but low PPV. This is perhaps unsurprising, as other clinical signs are easily mistaken for these classic diphtheria signs, particularly given the relative rarity of diphtheria under normal circumstances. The inclusion of additional signs and symptoms when screening on clinical presentation may serve to enhance the robustness of this procedure. This is particularly true in settings where widespread testing is not available and when a maximally sensitive screening case definition is desired, for example, during the latter stages of an outbreak in resource-poor settings.

Prior research has suggested that vaccination reduces diphtheria transmission by approximately 60%, which, when coupled with antibiotic treatment of symptomatic cases, can be enough to end an outbreak. However, in a population in which R_0_ is approximately 3.0, control can only be achieved with treatment of at least half of all symptomatic cases within 2 days of symptom onset in a fully immunized population [43]. The authors further suggest that among this population, more than three-quarters (78%) of symptomatic cases would need to be isolated and treated to end transmission and that a more feasible and effective strategy would be to instigate random, mass antibiotic administration. Meanwhile, improved delivery through routine vaccination and enhanced community engagement to address the various barriers to vaccination have been identified as key approaches to strengthen vaccine demand and acceptance among this population [63].

The observed decline in incidence after the end of the first round of the MVC suggests a possible role in vaccine-derived immunity in reducing transmission. However, while the recognized regimen for diphtheria vaccination requires at least a 6-month interval between the second and third doses in order to mount durable protection [15], there was just a 1-month delay between rounds 2 and 3 of the MVC in this setting. This may account for lower estimates of seroprotection at the ≥0.1 IU/mL cutoff (63% among 1 to 6 year olds and 77% among 7 to 14 year olds) than the reported vaccination coverage [4]. This might, in turn, explain the failure to completely interrupt transmission and eliminate the epidemic in the months following the vaccination campaign, with Rt oscillating at around 1 from January 2018 onwards. The remaining case clusters indicate that the interventions appear to have been insufficiently effective to interrupt transmission and eliminate the outbreak, further supported by the observed constant proportion of cases testing diphtheria positive.

There may be a need for earlier launching of the MVC with an expanded (older) target age group given the unusual age distribution of cases observed during this outbreak and its continuation despite the MVC. Consideration may also need to be given to shorter timings between doses coupled with additional rounds of campaign to address remaining gaps in immunity and possibly replacing the Td vaccine used among adults with a vaccine containing a higher dose of diphtheria toxoid. Enhanced detection and isolation of new cases by active case finding using community health workers, and more effective contact tracing, with possibly a greater proportion of contacts vaccinated and provided with prophylactic antibiotics, might have brought the epidemic to an earlier end. We found that vaccinated cases had a longer average delay from disease onset to presentation at health facilities, possibly as a result of reduced severity among vaccinated individuals, or of different health-seeking behavior among older individuals.

The limited global supply of both vaccine [50] and DAT [64,65] was an important consideration during this outbreak, particularly as there were multiple concurrent outbreaks. Although further details are not available, anecdotal reports suggest that the principal reason for those DAT-eligible patients not receiving DAT was concerns about access to a sustainable DAT supply, which led some healthcare providers to be more cautious with its administration, particularly during the peak of the epidemic. Although DAT is listed in WHO Model List of Essential Medicines [66], global access is increasingly difficult due to limited supply and decreasing demand, which has accompanied increasing vaccination coverage and an accompanying declining global incidence [57]. The Ad hoc working group for Diphtheria Antitoxin (DAT) was convened in November 2017 [67] and advocated and managed an increased global stockpile of DAT. Our findings support the need for an enhanced global supply of DAT, and further research to explore the feasibility and efficacy of treatment using reduced amounts of DAT, in light of the high proportion of side effects observed.

## Conclusions

This outbreak reminds us that diphtheria may still cause large, rapidly expanding outbreaks among susceptible populations in the vaccine era. An adequate global DAT stockpile needs to be maintained by an independent body, as for yellow fever, meningitis, and cholera vaccines [68]. Crisis-affected populations must have access to health services, including routine vaccination. The international community should advocate for these rights among neglected people, including the Rohingya population residing both in Myanmar and in Bangladesh.

## Supporting information

S1 Checklist Strengthening the Reporting of Observational studies in Epidemiology (STROBE) statement checklist, diphtheria outbreak, Cox’s Bazar, Bangladesh, November 10, 2017 to November 10, 2019. (DOCX)

S1 Analysis Plan Outline plan for data analytical approach, diphtheria outbreak, Cox’s Bazar, Bangladesh, November 10, 2017 to November 10, 2019. (DOCX)

## Abbreviations

CFR: case fatality ratio
CI: confidence interval
COVID-19: Coronavirus Disease 2019
DAT: diphtheria antitoxin
DTC: Diphtheria Treatment Center
EWARS: Early Warning, Alert and Response System
GCL: gross cervical lymphadenopathy
Hib: Haemophilus influenzae type b
IEDCR: Institute of Epidemiology Disease Control and Research
IQR: interquartile range
MOH&FW: Ministry of Health and Family Welfare
MSF: Médecins Sans Frontières
MVC: mass vaccination campaign
NPV: negative predictive value
PPV: positive predictive value
RR: risk ratio
Td: tetanus and diphtheria
UNHCR: United Nations High Commissioner for Refugees
WHO: World Health Organization
