# Supplementary material for: Epidemiological, clinical, and public health response characteristics of a large outbreak of diphtheria among the Rohingya population in Cox’s Bazar, Bangladesh, 2017 to 2019: A retrospective study
Source: PLoS Med. 2021 Apr 1;18(4):e1003587. doi: 10.1371/journal.pmed.1003587 (PMC8059831; doi:10.1371/journal.pmed.1003587)
Supplement: S1 Analysis Plan — (DOCX) [file pmed.1003587.s002.docx]

**Analysis plan for Diphtheria outbreak description, Rohingya population, Cox’s Bazar.**

**General objective**

Describe the outbreak of diphtheria among the Rohingya population in Cox’s Bazar, Nov 2017 onwards in terms of epidemiology, clinical characteristics, and response activities.

**Analytical plan**

All analyses should be restricted to Rohingya population and to confirmed, probable and suspected cases (i.e. excluding non-cases).

**A. Descriptive epidemiology**

1. Report case counts by population (Rohingya and host) and case definition, and their age and sex distributions. Use the following age groups: <7, 7-14, 15-29, 30-44, 45 and over (#/%)
2. Report outcomes – recovered, died, and lost-to-follow-up (#/%), and present the overall CFR
3. Report attack rates per unit person-years
4. Report the performance of contact tracing system as the proportion of contacts listed that were successfully traced, and the adherence to chemoprophylaxis at the three-day follow-up. (#/%)
5. Report the administrative coverage of the three rounds of mass vaccination campaign (%).

**B. Epidemiological parameters of the outbreak**

1. Estimate daily growth rates before and after epidemic peak, and provide estimates of doubling and halving times
2. Estimate the basic and time-varying reproduction numbers

**C. Clinical characteristics**

1. Descriptive epidemiology
   1. Explore delay distributions from dates of onset to report, to treatment, and to outcome
   2. Describe vaccination status among case-patients (#/%)
   3. Describe clinical characteristics of case-patients (#/%)
   4. Describe treatment characteristics of cases (#/%) – antibiotics and DAT
   5. Explore the sensitivity, specificity, PPV and NPV of the probable case definition (presenting with GCL or PM). Report as percentages. If these perform poorly, explore which additional signs/symptoms enhance the performance of the case definition.
2. Analytical epidemiology among confirmed cases
   1. Explore predictors for presenting with GCL and PM in unadjusted and adjusted multivariable Poisson regression models, with the following risk factors (keeping only those significant at the p<0.05 level for the adjusted analysis): age group; sex; vaccination status; and period of vaccination (campaign vs. pre-campaign)
   2. Explore predictors for risk of death in unadjusted and adjusted multivariable Poisson regression models, with the following risk factors (keeping only those significant at the p<0.05 level for the adjusted analysis): age group; sex; delay from onset to report; vaccination status; antibiotic and DAT treatment; and evidence/presence of respiratory distress, GCL and PM
   3. Explore predictors for testing positive for diphtheria in unadjusted and adjusted multivariable Poisson regression models, with the following risk factors (keeping only those significant at the p<0.05 level for the adjusted analysis): age group; sex; vaccination status; evidence/presence of GCL and PM; and period of vaccination (campaign vs. pre-campaign)

**D. Tables**

1. Demographics characteristics of case-patients, overall and by case-definition
2. Breakdown of signs and symptoms, complications and treatment outcomes, overall and by case definition
3. Performance of probable case definition against lab test, among those with lab test result
4. Results of Poisson regression for risk factors for cases presenting with GCL & PM
5. Results of Poisson regression for risk factors for dying
6. Results of Poisson regression for risk factors for testing positive

**E. Figures**

1. Epidemic curve by case definition
2. Epidemic curve showing timing of mass vaccination campaigns, with Poisson models fitted to the epidemic curve on both the growth and decline phases
3. Display the evolution of the time-varying reproduction number
